# Herbal Medicine for the Treatment of Vascular Dementia: An Overview of Scientific Evidence

**DOI:** 10.1155/2016/7293626

**Published:** 2016-12-27

**Authors:** Dennis Chang, Jianxun Liu, Kellie Bilinski, Li Xu, Genevieve Z. Steiner, Sai W. Seto, Alan Bensoussan

**Affiliations:** ^1^National Institute of Complementary Medicine, Western Sydney University, Penrith, NSW 2751, Australia; ^2^Xiyuan Hospital, China Academy of Chinese Medical Sciences, Chaoyang District, Beijing, China

## Abstract

Dementia is a leading cause of mental and physical disability. Vascular dementia (VaD) is the second most common cause of dementia after Alzheimer's disease (AD) constituting 10–15% of the dementia population. Currently there are no approved pharmaceutical options for VaD and the conventional anti-AD therapies provide only modest, short-term relief of symptoms associated with VaD. Herbal medicines have been used for the management of dementia-like symptoms for centuries and may provide viable therapies for VaD due to their multicomponent and multitarget approach. This review is designed to provide an updated overview on the current status of herbal medicine research, with an emphasis on Chinese herbal medicine, for the treatment of VaD or dementia. A case study is also provided to demonstrate the development process of a novel standardized complex herbal formulation for VaD. The article reveals some preliminary evidence to support the use of single and complex herbal preparations for VaD and dementia. Multiple issues in relation to clinical and preclinical research have been identified and future research directions are discussed.

## 1. Introduction

Dementia is a syndrome associated with progressive impairments in memory and learning ability, cognitive skills, behaviour, activities of daily living, and quality of life. There are more than 47.5 million people with dementia worldwide and 7.7 million new cases are added to the dementia pool each year [1]. In Australia, there are over 353,800 people living with dementia and the number is estimated to increase to 900,000 by the year of 2050 [2]. Dementia has surpassed cerebrovascular disease and lung cancer to become the 2nd leading cause of death in Australia [3].

There are numerous types of dementia, among which vascular dementia (VaD) is the second most common cause after Alzheimer's disease (AD). Other common forms of dementia include Parkinson's disease, dementia with Lewy bodies, frontotemporal dementia, Huntington's disease, and alcohol-related dementia. VaD is associated with cerebrovascular and cardiovascular diseases and constitutes 10–15% of dementia cases in western countries. In developing countries, the prevalence of VaD is higher, accounting for around 30% of the dementia prevalence, which is partially due to poorer control of cardiovascular risk factors [4]. VaD often coexists with other forms of dementia especially AD. Indeed, postmortem studies reveal that over 40% of clinically diagnosed VaD cases also have AD type of neurodegenerative pathology, which is the most common type of mixed dementia [5].

Currently cholinesterase inhibitors and glutamate receptor antagonists are the most effective pharmaceutical options for the treatment of AD [6]. These medications have also been used off-label in some countries for the symptomatic relief in people with VaD, but the safety and the long-term therapeutic benefits of these interventions in VaD remain uncertain.

In the absence of satisfactory pharmacological therapies, many people with VaD or dementia and their carers turn to complementary medicine. The common complementary medicine interventions for VaD and dementia and dementia risk-reduction include herbal medicine, acupuncture, nutraceuticals, yoga, tai chi, and music therapy. The use of herbal medicine for the treatment of ageing-related disorders was documented in the literature more than 2000 years ago in ancient China where herbal remedies were used to boost memory function and increase longevity [7]. Early preclinical and clinical evidence exists to support the use of herbal medicines either as single herbal preparations or as complex herbal formulations for VaD. This review paper aims to provide an updated overview of evidence to support some of the commonly used herbs and herbal combinations with an emphasis on Chinese herbal medicine for the treatment of the disease. Issues and challenges associated with herbal medicines are discussed, and a case study is provided to demonstrate the development process of a novel complex herbal formulation for VaD that takes advantage of modern pharmaceutical and pharmacological technologies.

## 2. Pathophysiology and Therapeutic Options for Vascular Dementia

Cognitive impairment (especially executive dysfunction) is the primary symptom of VaD, which can also cause a disturbance in mood and behaviour and reduce of quality of life. According to the blood vessels involved and the pathological processes, VaD can be divided into large vessel dementia (multiple infarcts or multi-infarct dementia), small vessel dementia (small vessel disease and microinfarction), strategic infarct dementia, hypoperfusive dementia, dementia related to angiopathies (hypertension, amyloid), haemorrhagic dementia, and familial vascular dementia.

**Figure 1:**
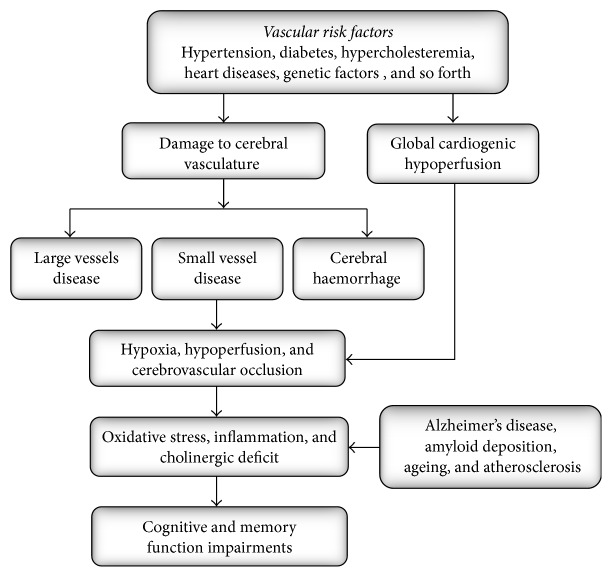
Pathophysiological mechanisms for vascular dementia.

The main risk factors associated with VaD include hypertension, hyperlipidemia, diabetes, genetic disposition, cardiac diseases, physical inactivity, and obesity [8]. The pathophysiology of VaD is complex. It incorporates interactions between vascular aetiologies (cerebrovascular disorders and vascular factors), changes in the brain (infarcts, white matter lesions, and atrophy), and host factors (age, education) [9]. The final common aetiopathogenic pathway usually attributes to a hypoxic, hypoperfusive, or occlusive process resulting in ischemic damage in various areas of the brain with subsequent cognitive and memory function impairments (Figure 1) [10]. Other pathogenic factors such as AD, amyloid deposition, ageing, and atherosclerosis also contribute to VaD development* via* inflammation and oxidative stress [8].

Currently, effective pharmaceutical interventions for VaD are lacking. Standard treatment largely focuses on symptomatic management and prevention of additional brain damage via recognition and control of cardiovascular and cerebrovascular risks using, for example, antihypertensives, aspirin, statins, vascular care, antidiabetes, and lifestyle modification [11]. Several classes of anti-AD pharmaceutical agents are used off-label for symptomatic management in VaD. Cholinesterase (ChE) inhibitors (donepezil, galantamine, and rivastigmine) and NMDA receptor antagonists (memantine) have shown some modest short-term clinical benefits in improving cognitive function; however, most of these studies fail to demonstrate significant improvements in global functioning, activities of daily living, and quality of life [12]. The majority of studies conducted so far are over a relatively short duration (5-6 months); therefore the long-term benefits and safety of these interventions in VaD have not been validated.

## 3. Individual Herbs Used in VaD

There is a long history of herbal medicine use to boost memory and cognitive functions and manage behavioral and psychological symptoms associated with dementia/VaD. Some of the most commonly used and studied herbs include* Ginkgo biloba*,* Huperzia serrata*,* Curcuma longa*,* Panax ginseng, Panax notoginseng, Bacopa monnieri, Salvia miltiorrhiza, Crocus sativus, *and* Camellia sinensis*.  Table 1 summarises the nomenclature, key bioactive compounds, and mechanisms of action of these herbs.

### 3.1. *Gingko biloba*

*Ginkgo biloba* leaf extract (ginkgo) is one of the most studied medicinal herbs. Ginkgo leaf extract is widely used for ageing-related memory disorders in many European and Asian countries. The principal constituents of ginkgo include flavonol glycosides (e.g., quercetin and kaempferol) and terpenoids (e.g., ginkgolide and bilobalide) [13]. Preclinical studies suggest that ginkgo decreases oxygen radical discharge and proinflammatory functions of macrophages (antioxidant and anti-inflammatory), reduces corticosteroid production (antianxiety), and increases glucose uptake and utilisation and adenosine triphosphate (ATP) production [14]. Ginkgo also appears to improve blood flow through increasing red blood cell deformability and decreasing red cell aggregation, inducing nitric oxide production, and antagonising platelet activating factor receptors [14]. EGb761 (a standard ginkgo preparation) treatment was also shown to enhance proliferation of neural stem cells in subventricular zones and the dentate gyrus [15] and to accelerate the recovery of the pathological synaptic plasticity [16] in VaD models in rats. In an ageing rat model, EGb761 reduced circulating free cholesterol and brain *β*-amyloid precursor protein production [17].

In animal studies, the effects of ginkgo leaf extracts on neuroprotection and cognitive dysfunctions have been demonstrated in various cerebral ischemia models in rats [18, 19], mice [20], and gerbils [21, 22]. In a recent study in rats with two-vessel (bilateral common carotid arteries) occlusion-induced VaD model, treatment of bilobalide significantly improved the learning and memory ability of the animals in a Morris water maze task [23].

In healthy young adults, ginkgo treatment has been shown to improve speed of processing, working memory, executive function, and cognition [24]. In a study with 80 patients with vascular cognitive impairment (not diagnosed with dementia), a combined therapy of ginkgo extract with conventional treatment of aspirin over three months significantly improved executive function, attention, abstract-thinking, delayed memory, and orientation when compared with the control group (aspirin treatment only) [25].

The evidence to support the use of gingko for dementia remains controversial. Although some clinical studies fail to show a significant difference between ginkgo and placebo in dementia groups [26], numerous clinical trials demonstrate that ginkgo improves memory loss and concentration and decreases anxiety in patients with dementia and/or VaD. For example, a randomised, double blind, placebo-controlled trial of 216 participants with AD or vascular dementia showed a significant improvement in attention and memory function in the EGb761-treated group after 24-week treatment [27]. In a more recent trial, 404 people with dementia (333 AD and/or mixed dementia and 71 VaD) were treated with 240 mg EGb 761 or placebo over 24 weeks. The results demonstrate that gingko treatment significantly improves cognitive function and neuropsychiatric symptoms [28]. No difference was found between the AD and VaD groups. These effects have been confirmed by several meta-analyses, indicating that ginkgo treatment stabilises or slows decline in cognition, function, and behaviour [26, 29, 30]. A recently published systematic review, in which nine relatively high quality clinical trials (six studies included participants with VaD or mixed dementia) were recruited, reported that EGb761 not only enhances scores of neurocognition but also improves activities of daily living in patients with AD and/or VaD/mixed dementia [30].

### 3.2. *Huperzia serrata*

*Huperzia serrata* has a long history in Chinese medicine for use in conditions including strains, swellings, schizophrenia, myasthenia gravis, and organophosphate poisoning. The key bioactive components of* Huperzia serrata* belong to the lycopodium alkaloids family including huperzine A (HupA), huperzine B (HupB), hyperzinine, carinatumin A, and carinatumin B, all of which possess antiacetylcholinesterase properties [31]. In particular, HupA became known globally after the discovery in the 1980s for its use as a potent acetylcholinesterase (AChE) inhibitor in the treatment of dementia. In addition, HupA has also been shown to exert other pharmacological effects including antioxidant, anti-inflammatory, antiapoptosis, anti-*β*-amyloid peptide fragmentation, inhibition of oxygen-glucose deprivation, and NMDA receptor antagonism [32, 33] (Table 2).

The majority of clinical trials of HupA to date have been conducted in China and have investigated its effect in AD patients. A meta-analysis of HupA for the treatment of AD identified 11 studies (one open-label study, two case reports, and eight controlled clinical trials), among which 4 trials involving 474 patients (235 in the HupA treatment group and 239 in the control group) were included in the final analysis [34]. The results demonstrate that HupA (300–500 *μ*g/day) significantly improves cognitive function, as assessed by the mini-mental state examination (MMSE) and activities of daily living (ADLs). Similarly, a recent Cochrane systematic review of six clinical trials with a total of 454 patients concluded that HupA treatment led to an improvement in general cognitive function, global clinical status, behavioral disturbance, and functional performance, with minimal side effects in AD patients [35]. Another systematic review and meta-analysis of 20 RCTs involving 1823 participants showed beneficial effects of HupA (200–800 *μ*g/day) on improvement of cognitive function, as measured by MMSE, Hasegawa Dementia Scale (HDS), and ADL [36], although the authors note that findings should be interpreted with caution due to the poor methodological quality of the included trials.

Among studies which have included VaD patients, one Cochrane review investigated HupA for VaD but only identified one small study involving 14 participants in which HupA was found to be no better than placebo [37]. A subsequent meta-analysis of placebo-controlled RCTs of HupA on patients with AD and VaD identified eight AD and two VaD trials with 733 and 92 participants, respectively [38]. HupA treatment (100–500 *μ*g/day) was shown to significantly improve the MMSE and ADL scores of AD and VaD patients, and longer treatment duration resulted in improved efficacy for AD patients. However, as noted in these reviews, the lack of quality data, small sample sizes of individual clinical trials, and short intervention periods limit firm conclusions about HupA's clinical efficacy, highlighting the need for rigorous randomised controlled trials with large sample sizes.

### 3.3. *Curcuma longa*

*Curcuma longa* (turmeric) is a food spice and colouring agent used in Chinese, Hindu, and Ayurvedic medicine for centuries has been applied in therapeutic preparations to treat numerous conditions such as pancreatitis, arthritis, cancer, and inflammatory, neurodegenerative, and digestive disorders. Curcumin and curcuminoids are the key bioactive components of turmeric consisting of three structurally closely related chemical components: curcumin, demethoxycurcumin, and bisdemethoxycurcumin [39]. Data from animal and/or* in vitro* studies suggests that curcumin can affect multiple pathological targets associated with dementia via inhibiting lipid peroxidation, scavenging reactive oxygen species (ROS), and reactive nitrogen species, inhibiting NF-kB activation, and its anti-inflammatory actions [31, 40]. It has also been suggested that curcumin is able to directly bind small beta-amyloid species to block aggregation and fibril formation [41].

Animal studies have shown that curcumin offers protective effects against VaD by exerting antioxidant and anti-inflammatory effects. A lower prevalence of AD in some Asian populations has been attributed to a curcumin-rich diet. One population-based study of 1,010 Asian seniors without dementia showed that consumption of turmeric containing curry was associated with improved cognitive function as measured by MMSE [42]. Based on these findings several clinical trials have been initiated [43–45]. One pilot randomised, double blind, placebo-controlled trial evaluated the pharmacokinetics and effects of curcumin supplementation (1–4 g/day) over six months in 34 AD patients [44]. The results of the study showed slight improvements in MMSE scores without significant side effects; however interpretation of these findings is limited due to the small sample size, short follow-up period, and lack of cognitive decline in the placebo group. In another 24 months, randomised controlled trial in 36 patients with mild-moderate AD and 2,000 mg and 4,000 mg/day of curcumin C3 Complex® over 24 weeks failed to demonstrate any clinical or biochemical evidence of efficacy of curcumin in AD [46]. The lack of positive findings may be somewhat attributed to curcumin's relatively low solubility and bioavailability. Further studies are required focusing on the active components of curcumin to determine the therapeutic value of curcumin in the treatment of dementia.

### 3.4. Ginseng

*Panax ginseng* (Ren Shen) and* Panax notoginseng* (San Qi) are two important members of the ginseng species and have been used for centuries in Chinese medicine to treat atherosclerosis, hypertension, thrombosis, external injury, and pain. In addition, ginseng has shown therapeutic benefits for learning and memory and may be useful in developing supplements for the prevention or potential treatment of AD [47, 48]. The principal bioactive components of ginseng are ginsenosides (e.g., ginsenosides Rg1, Rg3, and Rg5), which have been suggested to have antioxidant, anti-inflammatory, and antiapoptotic effects [49]. In addition, ginsenoside Rg5 has been shown to reduce amyloid-*β* and cholinesterase activity [50], while ginsenoside Rg3 has also been shown to promote *β*-amyloid peptide degradation via enhancing gene expression [51–54]. In addition, research demonstrates that* Panax ginseng* decreases blood pressure and improves blood circulation via vasodilation activities [55].

*Ginseng* is widely used to treat dementia-like symptoms in many Asian countries; however the majority of studies examining its effects on cognition have been studied in animals and healthy individuals. Clinical trial data suggests that ginseng modestly improves thinking and working memory in healthy volunteers. [56, 57]. Two open-label trials showed that 12-week treatment with ginseng improved AD Assessment Scale-Cognitive Subscale (ADAS-cog) scores in AD participants [58, 59].

Two recent small open-label trials demonstrate the potential therapeutic benefits of* Panax ginseng* for AD [59, 60]. In the former study, which showed significant effects on ADAS-cog and Clinical Dementia Rating (CDR) following 24-week treatment of low or high dose (4.5 g or 9 g/day)* Panax ginseng* compared to controls [58], subjects were followed up for further 2 years during which time cognitive function was evaluated every 12 weeks using the ADAS and the Korean version of the MMSE (K-MMSE). In the long-term efficacy evaluation of the effect of* Panax ginseng*, cognitive function was sustained for the follow-up period. In the latter study, in which 87 AD participants (58 in the ginseng group and 39 in the control group) were involved, 12-week treatment with* Panax ginseng* powder (4.5 g/day) produced significant improvements in ADAS-cog and MMSE scores [59]. Clinical benefits have also been demonstrated after* Panax ginseng* is combined with ginkgo in improving cognitive function in healthy subjects [14, 24, 61–63].

Less research has been conducted on* Panax notoginseng.* One randomised controlled trial in 40 people with VaD, which compared the effect of 12-week supplementation with* Panax notoginseng* to duxil, a drug that increases oxygen in brain tissue, showed that memory function significantly improved in those given the herb [64]. In a trial with 64 older adults with lacunar infarction (cerebrovascular disease), the effects of the injectable form of* Panax notoginseng* extract (Xueshuantong) were investigated. Four-week treatment of Xueshuantong significantly increased relative cerebral blood flow and improved the ADL scores, although MMSE scores showed no marked changes [65]. Large scale, long-term studies using standardized extracts are required to confirm the clinical efficacy of ginseng therapy in dementia and VaD.

### 3.5. *Bacopa monnieri*

*Bacopa monnieri* (Brahmi) has been traditionally used in Ayurvedic medicine to treat conditions including pain, asthma, fever, inflammation, and memory decline [66]. Various mechanisms may be involved in the neuroprotective and memory enhancing effects of Brahmi such as increasing antioxidant activity [67], free radical scavenging [68], binding and detoxification of metal ions [69], modifying levels of acetylcholine [68], and increasing cerebral blood flow via vasodilation [70]. The constituents responsible for improving learning and memory are attributed to steroidal saponins and bacosides A and B [71]. Bacosides enhance kinase activity and neuronal synthesis, which is linked with the restoration of synaptic activity, ultimately improving nerve impulse transmission [72].

Brahmi improves motor learning, acquisition, and retention and delays extinction of newly learned behaviour in animals [73]. A series of clinical studies have demonstrated the acute and chronic neurocognitive effects of Brahmi in healthy elderly populations [74]. A systematic review of six randomized controlled trials using a dose of 300–450 mg Brahmi per day showed that the compound preferentially enhances secondary memory, although the duration of supplementation in these trials was inadequate to substantiate the effects of Brahmi on cognition [75]. The Australian Research Council Longevity Intervention (ARCLI) study, a randomised, double-blind, placebo-controlled, 3-arm parallel-group clinical trial is currently underway in attempt to overcome this issue by examining the effect of 12-month administration of Brahmi on cognitive performance in 465 healthy participants [76]. A study to assess the effects of 6-month treatment of CDRI 08, a standardized Brahmi extract on cognitive function in AD patients, is also being planned by the same team [74].

### 3.6. *Crocus sativus*

*Crocus sativus* (Xi Hong Hua) commonly known as saffron is used in Chinese medicine as antidepressant, antispasmodic, and anticatarrhal. Data from* in vivo* and* in vitro* studies demonstrate that saffron possesses anti-inflammatory, antioxidant, and antiapoptotic properties [77]. Saffron extract has been shown to improve learning and memory function in ethanol-induced memory impairment in mice and to ameliorate cerebral ischaemia induced oxidative damage in the rat hippocampus [78, 79]. Crocin, the principal constituent of saffron and a strong antioxidant, has been suggested to be largely responsible for saffron's protective effect on the central nervous system [80]. It has also been suggested that saffron can act as an antidepressant and antiplatelet agent [81], both of which may offer additional benefits for people with dementia/VaD.

In more recent years, saffron has been used for neurological conditions [82]. A 22-week double-blind RCT in AD participants showed that saffron (30 mg/day) resulted in comparable improvements in cognition to donepezil (10 mg/day) although better tolerated [83]. Another 16-week double-blind trial in AD participants, comparing the same dose of saffron with placebo, showed saffron supplementation resulted in significantly better outcomes in cognitive function than placebo [83, 84]. These findings support the need for larger trials over a longer duration.

### 3.7. *Camellia sinensis*

*Camellia sinensis*, commonly known as tea, is widely consumed as a health beverage, especially in the form of green tea in Asia. The chief bioactive components of tea are polyphenols, caffeine, and amino acids. Catechin polyphenol constituent, epigallocatechin-3-gallate (EGCG), is the most potent bioactive component of green tea. ECCG been shown to exert neuroprotective/neurorescue activities via a wide range of mechanisms including downregulation of proapoptotic genes, elevation of *α*-secretase activity, and inhibition of *β*-secretase activity, anti-inflammation, scavenging of ROS, and stabilisation of mitochondrial function [31, 85, 86].

Animal and epidemiological studies have suggested that drinking green tea confers protection to the brain against the ageing process. An inverse correlation between tea consumption and the incidence of AD and other neurodegenerative diseases has been suggested [86], although longitudinal and cross-sectional studies investigating the effect of green tea on cognitive function have produced mixed findings.

A cross-sectional study assessing the effect of green tea consumption on cognitive function in 1,003 Japanese participants aged over 70 years showed that daily consumption of two or more cups of green tea (100 mL/cup) was associated with a lower prevalence of cognitive impairment [87]. A recent double-blind, counterbalanced, within-subjects study which compared the effect of 27.5 g green tea extract on working memory in 12 healthy subjects showed that green tea increased working memory, suggesting that green tea may be effective for the treatment of cognitive impairments in disorders such as dementia [88]. Another prospective population-based study of 723 Japanese participants with normal cognitive function at baseline found that the incidence of dementia was significantly lower in those who consumed green tea 1–6 days/week compared to those who did not consume green tea [89]. In contrast, a double-blind randomised controlled study assessing the effects of green tea consumption (2 g/day) in 33 nursing home residents with cognitive dysfunction was unable to show a significant improvement in cognitive function as assessed by MMSE [90].

In summary, multiple herbs have demonstrated potential therapeutic benefits for improving cognitive function in dementia and/or VaD. However, most of the evidence comes from preclinical research, and many of these findings have not been directly validated in people with VaD. In addition, plasma concentrations of the bioactive components of these herbs are generally below the levels able to generate meaningful pharmacological activity. One possible explanation for these clinical effects is that these bioactive components interact synergistically leading to greater pharmacological/clinical outcomes than predicted by the activity of individual components. However, the evidence to support this theory is generally lacking and further research is needed to assess synergism of herbal medicine, as detailed below in Section 4.1.

## 4. Complex Herbal Formulations

### 4.1. Mechanisms and Synergistic Effects

Multiple herbs are often combined in complex formulations in some traditional medical systems for the treatment of various diseases. Theoretically, this multicomponent and multitarget approach may be ideal for diseases that have complex aetiologies and pathophysiologies such as VaD.

In TCM, the use of multiherbal therapies in which up to 20 herbs are used underpins its unique philosophy and holistic approach. According to TCM theories, multiple herbs are included in a complex herbal formulation based on the principle of “Jun (emperor) - Chen (minister) - Zuo (assistant) - Shi (courier).” The “Jun” herb is the key therapeutic component of the formula directly targeting the disease, the “Chen” herb is included to relieve the accompanying symptoms of the disease and/or to enhance the effects of the key herb, the “Zuo” herb reduces toxicity of the herbal formula, and the “Shi” herb facilitates the delivery of active components of the formula to the target organs and/or harmonises their effects.

The Jun-Chen-Zuo-Shen theory describes a complex interactive relationship where the herbs in a complex herbal formulation interact synergistically to enhance distribution and/or ameliorate/prevent potential side effects. Some of these interactions are able to be explained pharmacologically. For example, bioactive components in herbal formulations interact improving their solubility and subsequent bioavailability, enabling them to affect multiple therapeutic targets associated with the disease and/or to enhance metabolism of toxic components thereby reducing side effects [91].

Evidence to support these beneficial interactions is very limited and results remain controversial. The paucity of data is partially caused by the lack of robust research methodologies to study the synergistic effects of multicomponent herbs or herbal formulations. The two methods most commonly used for studying synergism are the combination index (CI) and isobole method. Both methods have been developed to determine synergistic or antagonistic interactions between two or more single-entity agents acting on the same target/receptor and require the determination of a dose-response relationship of the combination and its individual components [92]. However, these methods are inadequate for evaluation of synergy in complex herbal formulations where multicomponents interact with multiple therapeutic targets/receptors [93].

The use of the systematic analysis or system-to-system (S2S) method is gaining momentum in the study of multitarget synergistic actions. Taking advantage of computational sciences, the S2S approach integrates data from literature and experimental studies. S2S analysis is conducted via a docking process during which three-dimensional structures of individual compounds of interest are matched against the known structures of relevant key therapeutic protein targets associated with the disease using computer software. However, there is general lack of information on the chemical and pharmacologic properties of bioactive components of many herbal medicines and therefore the use of the S2S method in the study of complex herbal formulations remains a challenge [92].

### 4.2. Clinical Evidence of Complex Herbal Formulations for VaD

The current clinical evidence to support the use of complex herbal formulations for dementia and/or VaD remains weak and controversial. There are only a limited number of reports published in English that examine the effectiveness of complex herbal formulations for VaD. For example, in a randomised, double-blind, placebo-controlled trial, the effects of a traditional Chinse herbal formulation,* Bai Wei Di Huang Wan* (consisting of 8 Chinese herbs), were examined in 33 patients with mild to severe dementia. Although the authors did not specify the types of the dementia, 91% of the patients recruited exhibited neuroimaging evidence of cerebrovascular disease and therefore it is likely that these patients had VaD or mixed dementia. The authors found that 8-week treatment of* Bai Wei Di Huang Wang* formula significantly improved cognitive function (measured by MMSE) and ADL (measured by Barthel Index) when compared to the placebo [94]. However, the trial was not fully powered and the invention period was relatively short. Further studies with larger sample sizes and longer duration are required to confirm these results.

The effects of a seven-herb Kampo/Chinese medicine formula,* Yokukan-san* (*Yi-Gan San* in TCM) on neurocognitive function and behavioral and psychological symptoms, were investigated in an open-label trial in 13 people with VaD according to the National Institute of Neurological Disorders and Stroke-Association Internationale pour la Recherche et l'Enseignement en Neurosciences (NINDS-AIREN) diagnostic criteria. Yokukan-san consists of* Angelica acutiloba*,* Atractylodes lancea* rhizome,* Bupleurum* radix,* Poria sclerotium*,* Glycyrrhizae radix*,* Cnidium* rhizome, and* Uncarie* hook. Although 4-week treatment of* Yokukan-san* did not significantly change the MMSE scores, there was a significant change in the overall NPI (neuropsychiatric inventory) score and mean subscores for agitation and disinhibition after the treatment, suggesting potential neuropsychiatric benefits of the formula [95].

A large number of trials were conducted in China to evaluate various complex Chinese herbal formulations for the treatment of VaD. A systematic review published in 2012 detailed 47 randomised controlled clinical trials (all conducted in China) involving 3,725 people with VaD (using diverse diagnostic criteria) in an effort to assess the safety and efficacy of herbal medicines for VaD [96]. Out of 43 studies where herbal medicines were used as monotherapies, 37 reported that the herbal interventions exerted significantly greater effects than the conventional medicines (piracetam, aniracetam, hydergine, etc.) or placebos. All 4 studies in which herbal medicines were used in conjunction with conventional medicines reported better neurocognitive efficacy outcomes compared to the conventional medicines alone. However, significant methodological issues were identified in these studies including no sample size calculation, inconsistent diagnostic criteria used, differences in baseline characteristics, inappropriate randomisation, and diverse outcome measures used (some studies used instruments developed in-house). In addition, some 43 different herbal/complex herbal preparations were used in these studies. Each of these methodological shortcomings seriously impacts on the significance of these clinical findings.

A more recent meta-analysis conducted by Gong et al. [97] using strict inclusion criteria (e.g., exclusion of studies using single herbs or with short duration) included 24 randomised clinical trials comprising 2043 people with VaD (all conducted in China), although no analysis of the VaD diagnostic criteria used in these studies was provided. In a subgroup analyses, complex Chinese herbal interventions significantly enhanced cognitive function (judging by MMSE scores) when compared to piracetam (in 10 studies) or placebos (in 3 studies). No difference in MMSE scores was identified between the Chinese herbal medicines and hydergine in 17 of the studies. Yet, herbal medicine treatments produced a greater improvement in ADLs compared to piracetam treatment in 5 of the studies. Having said this, similar methodological problems to the previous systematic reviews were identified in these studies.

In summary, a number of complex herbal formulations have been trialled clinically but mainly in China. There are numerous methodological problems with most of these clinical trials. In addition, no studies reported the standardization of their herbal interventions used in the trials. Mechanistic studies to evaluate mechanisms of action and synergy of these formulations are also lacking.

## 5. SLT, a Case Study on the Development of a Complex Herbal Formulation for VaD

Recognising the lack of therapeutic options for the treatment of VaD, a combined team from the China Academy of Chinese Medical Sciences and Western Sydney University (authors of this article have contributed to the project in various ways) has been working together to develop a standardized complex herbal formulation, SLT (Sailuotong, previously known as WNK) for the treatment of VaD. SLT represents a new generation of herbal medicine whereby the chemical and pharmacological profiles have been clearly defined. Over the last 10 years, a substantial body of evidence has accumulated in support of the use of SLT for VaD. Some of the key data have been summarised in this section by way of providing an example to demonstrate the developmental process of new complex herbal interventions for the treatment of serious diseases such as VaD.

### 5.1. SLT Development Process

SLT formula consists of specific dosages of standardized* Ginkgo biloba* (ginkgo),* Panax ginseng* (ginseng), and* Crocus sativus* (saffron) extracts, designed for the treatment of VaD. The herbs were selected based on their traditional use and existing clinical and pharmacological evidence (as summarised in Sections 3.1, 3.4, and 3.6). A number of bioactive components have been identified in ginseng, ginkgo, and saffron that have a variety of pharmacological effects associated with VaD. The effects of these key bioactive components of SLT formula are summarised in Table 2.

**Figure 2:**
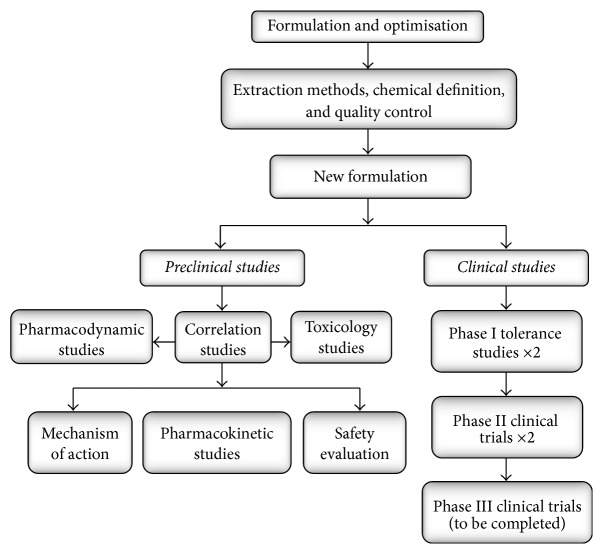
Development process of SLT, a novel, standardized complex herbal formulation for VaD.

In the development of SLT, bioassay-guided fractionations were used to determine optimal organic solvent-based extraction methods for each herb individually. The optimal dose ratio and dosage regimen were determined through a series of pharmacological studies using various animal models. These models include VaD/ischemia models (e.g., bilateral common carotid artery occlusion model in rats, MCAO-induced cerebral infarction in rats), acquired memory impairment models induced by D-galactose, scopolamine, reserpine, and alcohol, and platelets aggression model in rabbits. The development process of SLT is summarised in Figure 2.

Based on these studies, a new formulation was developed and standardized through a rigorous quality control program where 10 bioactive markers were quantified in the final product. The pharmacodynamic and pharmacokinetic properties of the formula were determined and the acute and chronic toxicity evaluated in animals. Human tolerability of the formula was determined in single administration and multiple (continuously) administration phase I studies and the clinical effectiveness, optimal clinical dose, and safety were determined in phase II studies. The efficacy and safety will be further evaluated in phase III studies where a greater number of VaD participants will be recruited.

### 5.2. Pharmacological Investigations of SLT

A series of preclinical pharmacokinetic and pharmacodynamic studies were conducted on SLT formula and its individual components. The data from these experiments demonstrated significant improvements in learning and memory function, pathogenic biochemical parameters in blood and brain tissue, and antioxidant capacity in various experimental dementia models.

In an* in vivo* study, the effect of SLT treatment (11, 22, and 44 mg/kg per day over 15 days) on memory impairment was investigated in acquired/consolidated dysmnesia models in mice induced by scopolamine, reserpine, chlorderazin, sodium nitrite, and alcohol. Compared with the control group, the medium and high dose treatments of SLT markedly decreased the error numbers and prolonged the latencies in all dysmnesia groups receiving active treatments, suggesting that SLT possesses beneficial protective effects on chemically induced cognitive impairments [98].

In a chronic cerebral hypoperfusion model induced by bilateral common carotid artery ligation in rats, 8-week treatment of SLT intragastrically (ig) significantly shortened the persistent time of finding the platform in a Morris water maze task [99]. Activity of cholinesterase was also significantly decreased (*p* < 0.05) while the acetylcholine (ACh) level was markedly increased in the brain tissue (*p* < 0.05). In addition, the activity of superoxide dismutase (SOD) was significantly enhanced (*p* < 0.05) [99]. These results suggest that SLT treatment improves hypoperfusion-induced cognitive impairments and this change may be associated with the cholinergic protective effect and free radical scavenging capacity of SLT formula.

The effects of SLT on ACh were also investigated in an amyloid *β*-protein induced dementia model in mice [52]. One-month treatment of SLT (15.5 mg/kg and 31.0 mg/kg per day, ig) significantly increased the ACh levels in the brain tissue by 18.6% and 20.0%, respectively, when compared with the model group. In another study, a longer treatment of SLT over 12 weeks at 31 and 62 mg/kg per day ig significantly increased the ACh levels in hippocampus in both treatment groups in a PDAPP^v7171^ transgenic dementia model in mice. In addition, hippocampal serotonin level was decreased significantly in the high dose SLT group [100].

The effects of crocin alone (the principal active component of* Crocus sativus*) on ischemia/reperfusion injury were investigated using a global or bilateral common carotid artery occlusion model in mice [101]. Pretreatment with 20 mg/kg crocin significantly inhibited oxidative stress (MDA content; *p* < 0.001) in mice with 20 min of arterial occlusion followed by 24 hours of reperfusion. This change was accompanied by a significant elevation in total antioxidant capacity (increased SOD and glutathione peroxidase).

SLT has also demonstrated a range of cerebrovascular benefits. Acute SLT treatment over 24 h (8.25, 16.5, and 33 mg/kg) decreased the areas of focal cerebral ischaemia/reperfusion injury in rats and increased cerebral blood flow was also observed 60–180 min after administration (10 mg/kg) [102]. Seven-day SLT treatment (16.5 and 8.25 mg/kg) also showed a decrease in platelet aggregation rate and whole blood viscosity in rats [102].

In a pharmacokinetic study of SLT in rats, four bioactive components of ginkgo including bilobalide B and ginkgolides A, B, and C were found in rat plasma after oral ingestion (60 mg/kg), with a half-life between 1.6 and 2.8 h [101]. These constituents (especially bilobalide B and ginkgolide A) were also found in brain tissue indicating that they were able to penetrate through the blood brain barrier into the brain tissue.

In an* in vitro* study, the antioxidant effect of SLT was investigated in cultured human vascular endothelial cell, EAhy926. SLT (1–50 *μ*g/mL) significantly suppressed the H_2_O_2_-induced cell death and abolished the H_2_O_2_-induced ROS generation in a concentration dependent manner comparable to gallic acid (10 *μ*g/mL), a well-known antioxidant [103]. In addition, SLT (1–50 *μ*g/mL) significantly suppressed H_2_O_2_-induced LDH release. These results demonstrate that SLT has strong antioxidative and antiapoptotic activities on vascular endothelial cells. These properties may be associated with the neurological protective effects of SLT observed in people with VaD.

### 5.3. Clinical Investigations of SLT

A phase 1 study of SLT was conducted in 54 participants for evaluation of tolerance and safety. In the single administration study (60–540 mg/dose, 30 participants), the following adverse events were observed: stomach discomfort, occurrence of urticaria, local skin pain, diarrhoea, itchy skin, dry mouth, heartburn, abdominal distension, dizziness, and nausea [104]. However, there was no significant difference in the proportion of these adverse events between the treatment group and the placebo group. Furthermore, the distribution of adverse events was not dose-related. In the continuous administration study, 24 healthy volunteers were randomised to receive a low (180 mg/day) and a high dose (300 mg/day) treatment of SLT or equivalent doses of placebos over 14 days. There was no significant difference in the incidence rate of adverse events between the continuous treatment group and placebo group. No abnormal SLT treatment-related changes in liver and kidney function or ECG were observed. Overall, the results of the study showed that SLT was safe and well tolerated.

Using a randomised, double-blind, placebo-controlled crossover design, the effect of 1-week of SLT treatment on neurocognitive and cardiovascular function in 16 healthy adults was assessed [105]. In comparison to placebo, treatment with SLT resulted in a trend towards improvement in neuropsychological measures of working memory (immediate recall and N-back tasks) and in the brain's electrical response when attended information is encoded in memory (nonsignificant increase in P3a amplitude and significant decrease in N1 amplitude). The study showed that short-term SLT treatment is associated with more efficient attentional processing of auditory information and increased activation of working memory processes, suggesting that SLT has the potential to improve working memory performance in healthy adults.

In a phase II randomised, double-blinded, placebo-controlled pilot clinical study, 62 patients (32 in active group, 30 in placebo group) with probable or possible VaD were recruited according to the NINDS-AIREN criteria. Patients received 16-week treatment of either active compound or identical placebo after randomisation. At completion of the treatment, mean reductions in scores of the primary efficacy parameter, ADAS-cog, were 4.18 ± 0.75 and 1.18 ± 0.58 in participants receiving SLT and placebo, respectively [106, 107]. Although there is a difference in ADAS-cog at baseline (statistically nonsignificant) between the two groups, analysis of covariance (ANCOVA) showed that the improvement was significantly greater in SLT group than those in the placebo group after controlling for baseline ADAS-cog scores. A mechanistic substudy tested brain blood flow in 18 patients (7 patients SLT; 11 patients placebo) using Single Photon Emission Computed Tomography (SPECT). It was found that SLT treatment appeared to increase blood flow to the inferior frontal and anterior temporal lobes; regions associated with memory function and auditory and speech processing. This increase was more marked on the left when compared with the baseline in the treatment group only [106, 107]. This study reported no serious adverse events.

A second phase II dose determination study of SLT (240 mg and 360 mg per day over 52 weeks) in 325 patients with probable VaD has recently been completed. The preliminary analyses of the data indicate that 12-month treatment with SLT significantly improved cognitive function. No serious adverse reactions were reported. The full study report and publication are currently under preparation.

In summary, there are a range of challenges facing the development of complex herbal formulations. These challenges include (but are not limited to) selection of appropriate herbs, development of robust extraction methods and appropriate bioassay models, control of batch-to-batch quality, consistency of final products, establishment of pharmacokinetic and pharmacodynamic properties and toxicity profiles, and evaluation of effectiveness and safety through rigorous clinical trials. The SLT project provides an excellent example to demonstrate how some of these issues can be addressed. A considerable body of preclinical and clinical evidence has been constructed to support the use of SLT in VaD. Further work is now underway to further validate the effectiveness and safety of SLT with a greater VaD population in two multicentre phase III clinical trials. The preliminary trials detailed above provide promising findings that if supported by the phase III studies may lead to a breakthrough treatment for VaD.

## 6. Summary and Conclusion

Preliminary evidence demonstrates the potential therapeutic benefits of herbal medicine either as single preparations or as complex herbal formulations for the treatment of dementia, VaD, or mixed dementia. However, much of the evidence comes from animal and* in vitro* studies and overall clinical evidence to support these herbal interventions especially complex herbal preparations remains weak.

Multiple methodological issues have been identified in existing clinical trials. In addition to the general problems associated with sample size calculation, randomisation process, and statistical analysis of the results, several dementia/VaD trial specific issues also exist. For example, many trials did not specify the diagnostic criteria used to define AD, VaD, and/or mixed dementia while, in other trials, diverse diagnostic criteria such as DSM-III, DSM-III-R, DSM-IV, ICD-10, ADDTC, HIS, and NINDS-AIREN were used. There is also a lack of consistency in the instruments used for assessment of cognitive function and some instruments that were used have not been validated in dementia and/or VaD cohorts.

The treatment durations in the existing trials are also of concern. Dementia is a progressive disease and therefore longer duration clinical trials are required to appropriately assess the effects of interventions on the disease progress. The guidelines on medical products for AD and other dementias published by the European Medicines Agency mandate that controlled clinical trials aiming at demonstrating short-term improvement in AD and VaD should last at least 6 months and 12 months, respectively [108]. In addition to cognitive functions, the changes in ADL, global clinical improvement, quality of life, neuropsychiatric symptoms, and carer burden should also ideally be investigated in the future trials.

Few mechanistic studies exist to assess the synergistic effects among the multiple herbs and/or multiple active components. This is by and large caused by the lack of robust methods to evaluate synergy. As such, more research is urgently needed in this area. Herbal medicine, particularly Chinese herbal medicine, has been widely used for the control of various risk factors such as hypertension, atherosclerosis, and diabetes associated with cardiovascular disease and VaD. However, no studies were identified that assessed the prevention of VaD using herbal medicines. A number of studies evaluating the conversion of mild cognitive impairment to AD using long-term ginkgo treatment failed to demonstrate positive outcomes [109, 110]. Future epidemiological and clinical studies are required to further assess the benefits of herbal medicines for the prevention of dementia and/or VaD.

In conclusion, the existing evidence to support the use of single and complex herbal preparations is promising but requires further development. There are numerous issues relating to the trial design in clinical studies of herbal medicines for dementia and VaD. The case study outlined here demonstrates the feasibility and potential of developing evidence-based herbal medicines for the treatment of VaD.

## Figures and Tables

**Table 1:** Nomenclature, key bioactive compounds, and mechanisms of action of commonly used herbs for VaD.

Botanic name	Chinese pinyin or other names	Key bioactive compounds	Possible mechanisms of action associated with VaD
*Ginkgo biloba* L	Yin Shing Ye, Ginkgo leaf, EGb761 (standardized ginkgo extract)	Quercetin, kaempferol, ginkgolides (e.g., ginkgolide B, ginkgolide C), and bilobalide	(i) Antioxidant via decreasing oxygen radical discharge (ii) Anti-inflammation via decreasing proinflammatory functions of macrophages (iii) Increase glucose uptake and utilization and ATP production (iv) Improve blood flow by increasing red blood cell deformability and decreasing red cell aggregation (v) Induce nitric oxide production (vi) Inhibit platelet activating factors

*Huperzia serrata*	She Zu Shi Shan, toothed clubmoss, huperzine A (key bioactive extract)	Huperzine A (HupA), huperzine B (HupB), hyperzinine, carinatumin A and B	(i) Potent antiacetylcholinesterase (ii) Antioxidant (iii) Antiapoptosis (iv) Anti-*β*-amyloid peptide fragment (v) Inhibition of oxygen-glucose deprivation (vi) MMDA receptor antagonism

*Curcuma longa*	Jiang Huang, turmeric, ginger yellow	Curcumin, demethoxycurcumin, bisdemethoxycurcumin, curcuminoids, turmerone, zingiberene	(i) Antioxidant *via* inhibiting lipid peroxidation, scavenging ROS, and reactive nitrogen species (ii) Anti-inflammation (iii) Block aggregation and fibril formation (iv) Cholesterol-lowering properties

Ginseng *(Panax ginseng, Panax notoginseng)*	Panax ginseng: Ren Shen, ginseng, Korean ginseng *Panax notoginseng*: San Qi, Sanchi, Chinese notoginseng	Ginsenosides Rb1, Rg1, Rg2, Rg3, Rg5, Rc, Rd, Re notoginsenosides R1, R2, R3	(i) Antioxidant (ii) Antiapoptosis (iii) Anti-inflammation (iv) Reduce amyloid-*β* and cholinesterase activity (v) Decrease blood pressure and enhance blood perfusion.

*Bacopa monnieri*	Brahmi	Bacosides (e.g., bacoside A, bacoside B, etc.) brahmine, nicotine, herpestine	(i) Antioxidant activity (ii) Free radical scavenging (iii) Increased cerebral blood flow via vasodilation (iv) Restoration of synaptic activity and improving nerve impulse transmission (v) Modifying ACh level (vi) Binding and detoxification of metal ions (metal chelation) (vii) Removal of *β*-amyloid deposits (viii) Antidepressant

*Crocus sativus*	Xi Hong Hua saffron	Crocin, crocetin	(i) Antioxidant effect (ii) Antiapoptosis (iii) Anti-inflammation (iv) Antidepressant (v) Antiplatelet aggregation

*Camellia sinensis *	Cha green tea	Polyphenols (e.g., epigallocatechin-3-gallate), caffeine, amino acids	(i) Antioxidant effect (ii) Antiapoptosis (iii) Anti-inflammation (iv) Elevation of *α*-secretase activity and inhibition of *β*-secretase activity

**Table 2:** Multitarget mechanisms underlying pharmacological effects of SLT components.

Therapeutic targets associated with VaD	Ginseng			Ginkgo			Saffron		
	Rg1	Re	Rb1	Rd	Ginkgo flavonoids	Ginkgolides	Total flavone-glycosides	Crocetin	Crocin
Excitatory amino acid			X	X	X	X	X		
Energy depletion						X	X		
Calcium overload			X	X		X	X		X
Inflammation cascade				X		X			
Oxidative stress	X	X				X	X		
Cholinergic system	X	X					X		
Apoptosis	X		X				X		
Cytoskeleton						X	X		
Antithrombus	X						X		
Fibrinolysis	X						X		
Platelet aggregation					X	X	X	X	X
Cerebral circulation	X				X		X		
